# Integrated multi-omics analysis reveals gut dysbiosis and altered energy metabolism in Chinese ALS patients

**DOI:** 10.1128/spectrum.00609-26

**Published:** 2026-04-30

**Authors:** Xueli Ma, Zewei Jiang, Tianhua Yang, Hao Zhang, Wei Lu, Kuai Liu, Xinfeng Fan, Gaoyi Yang, Shenghai Wu

**Affiliations:** 1 Department of Laboratory, Hangzhou Geriatric Hospital 845709 , Hangzhou, China; 2 Department of Laboratory, Affiliated Hangzhou First People's Hospital, School of Medicine, Westlake University 557712, Hangzhou, China; 3 The Fourth School of Clinical Medicine, Zhejiang Chinese Medical University, Hangzhou, China; 4 Department of Ultrasonography, Hangzhou Geriatric Hospital 845709 , Hangzhou, China; 5 Department of Ultrasonography, Affiliated Hangzhou First People's Hospital, School of Medicine, Westlake University 557712, Hangzhou, China; Cleveland Clinic Lerner Research Institute, Cleveland, Ohio, USA

**Keywords:** amyotrophic lateral sclerosis, gut microbiome, metabolomics, oxidative stress, gut-brain axis

## Abstract

Amyotrophic lateral sclerosis (ALS) is a fatal neurodegenerative disorder with a complex etiology. Emerging evidence implicates gut microbiota dysbiosis in ALS pathology via the gut-brain axis, yet the specific integrative profile of the gut microbiome, virome, and metabolome, particularly in Chinese patients, remains incompletely characterized. Although global diversity indices showed no significant differences, taxonomic analysis revealed distinct compositional shifts. The ALS microbiome was characterized by a significant depletion of beneficial anti-inflammatory genera, specifically *Akkermansia* and *Faecalibacterium*, and an expansion of opportunistic pathogens such as *Escherichia* and oral-associated taxa (e.g., *Streptococcus*). We also observed a specific alteration in the gut virome, with viral genera including *Puppervirus* and *Donellivirus* enriched in ALS patients. Functionally, the ALS microbiome exhibited a marked upregulation of pathways involved in L-ascorbate (vitamin C) degradation and fatty acid biosynthesis, suggesting a microbial contribution to systemic oxidative stress. Metabolomic analysis corroborated these findings, identifying 271 differentially expressed metabolites. ALS patients showed elevated levels of inflammatory lipids (e.g., LysoPC) and metabolic intermediates of the tricarboxylic acid (TCA) cycle, alongside a downregulation of antioxidants. Integrative analysis highlighted profound dysregulation in porphyrin metabolism, oxidative phosphorylation, and energy homeostasis. Our findings demonstrate that ALS is associated with a specific dysbiotic gut ecosystem characterized by the loss of protective commensals, unique viral signatures, and functional metabolic reprogramming that exacerbates host oxidative stress and energy deficits. These results provide new insights into gut-brain interactions and highlight microbial antioxidant depletion as a potential therapeutic target.

**IMPORTANCE:**

Amyotrophic lateral sclerosis (ALS) is a devastating disease with no cure. While gut bacteria are known to influence brain health, we still do not understand exactly how they contribute to ALS progression. In this study, we used advanced DNA sequencing and chemical analysis to deeply examine the gut ecosystem of ALS patients. Beyond just cataloging which bacteria are present, we discovered what they are doing: the ALS microbiome actively breaks down vitamin C (a critical antioxidant) and disrupts energy metabolism. We also found a loss of protective bacteria that maintain the gut barrier. These findings are significant because they suggest that the gut microbiome in ALS patients may be actively fueling the disease by depleting the body’s antioxidant reserves. This points to a new potential treatment strategy: targeting these specific bacterial functions or replenishing specific metabolites to protect motor neurons.

## INTRODUCTION

Amyotrophic lateral sclerosis (ALS) is a fatal, progressive neurodegenerative disease that primarily affects motor neurons in the cerebral cortex, brainstem, and spinal cord. It leads to muscle atrophy, weakness, and paralysis, ultimately resulting in death from respiratory failure (1). Its etiology is complex, involving multiple mechanisms including genetics (such as mutations in SOD1 [2] and C9orf72 [3]), environmental factors, oxidative stress, mitochondrial dysfunction, neuroinflammation, and protein misfolding (2–4). However, the exact pathogenesis remains incompletely understood, and there is currently no effective cure. Numerous studies have shown that the composition and function of the gut microbiome (a condition known as dysbiosis) are closely related to the development and progression of various neurological diseases (5), including Parkinson’s disease (6), Alzheimer’s disease (7), autism (8), and multiple sclerosis (9). Therefore, an integrative analysis investigating the potential role of the gut microbiome and metabolome in ALS is crucial.

In recent years, the concept of the “gut-brain axis” (10) has gained wide acceptance. This axis describes a complex, bidirectional communication network between the gut microbiota, the enteric nervous system, the immune system, and the central nervous system. Gut microbes can affect the brain via multiple pathways: producing neuroactive substances (such as short-chain fatty acids [SCFAs] [11], neurotransmitter precursors [12], and tryptophan metabolites [13]), regulating immune responses (14) (e.g., affecting microglia activity and cytokine release), compromising gut barrier integrity (“leaky gut” leading to systemic inflammation), transmitting signals directly through the vagus nerve, and modulating host metabolism (15).

Previous studies have increasingly linked alterations in the gut microbiota to ALS. Fecal microbiota transplantation has shown potential in improving respiratory symptoms in ALS patients (16, 17), and probiotic supplementation can enhance motor function in murine models (18). Despite these findings, therapeutic options remain limited, with only riluzole and edaravone approved by the FDA (17, 19), offering modest benefits. Furthermore, most existing microbiome studies in ALS have relied on 16S rRNA amplicon sequencing, which offers limited taxonomic resolution and lacks functional genomic information. Critically, the gut microbiome is highly influenced by geography and dietary patterns, yet integrative multi-omics data regarding the gut microbiome and metabolome in Chinese ALS patients remain scarce. It remains unclear how specific bacterial species interact with host metabolic pathways to influence disease pathology. Therefore, a comprehensive analysis combining high-resolution metagenomics and metabolomics is urgently required to unravel these complex gut-brain interactions.

In the present study, we addressed these limitations by conducting a comprehensive integrative analysis utilizing metagenomic shotgun sequencing and LC-MS-based untargeted metabolomics in a cohort of Chinese ALS patients. Unlike previous studies primarily relying on 16S rRNA sequencing, our approach enabled a deep characterization of the gut ecosystem at the species level, alongside a detailed profiling of functional genes and fecal metabolites. We systematically evaluated the multidimensional alterations in the gut microbiome-metabolome interface and explored the potential mechanistic links between specific gut taxa and host metabolic dysregulation (e.g., oxidative stress and energy homeostasis). This work aims to provide a high-resolution reference for the specific microbiota-metabolic signatures of ALS, potentially unveiling novel non-invasive biomarkers and actionable targets for future therapeutic interventions.

## MATERIALS AND METHODS

### Study participants

This study enrolled 11 patients with ALS and 11 age-matched and sex-matched healthy controls (HC) in China. The demographics of the participants are shown in Table 1. ALS patients were screened for enrollment according to the revised El Escorial criteria, based on clinical presentation, electrophysiological findings, imaging, and genetic testing. Patients with gastrointestinal diseases, diabetes, hypertension, inability to eat normally, or severe illness requiring respiratory support were excluded. None of the participants had a history of diseases or recent antibiotic usage that could significantly alter their gut microbiota.

**TABLE 1:** Characteristics of study participants^*a*^

Characteristics	ALS^*b*^	HC^*c*^
*n*	11	11
Age (years)	51.8 ± 3	51.5 ± 5
Gender, M/F	5/6	5/6
Bulbar	1	N/A^*e*^
Upper limb (UL)	6	N/A
Lower limb (LL)	4	N/A
ALSFRS score^*d*^	35.82 ± 6.84	N/A
ROADs score^*d*^	36.45 ± 9.38	N/A
Average duration (in months)	26	N/A
BMI (kg/m^2^)	23.59 ± 3.16	24.67 ± 3.53
Diabetes	N/A	N/A
Dieting	N/A	N/A
Gastrointestinal tract diseases	N/A	N/A
History of gastrointestinal surgery	N/A	N/A
Enteral or parenteral nutrition	N/A	N/A
Non-invasive ventilation	N/A	N/A
Trauma in past	N/A	N/A

^*a*^ Most of the data are presented as mean ± SD.

^*b*^ Definite clinical diagnosis of ALS patients according to revised El Escorial criteria.

^*c*^ Healthy controls.

^*d*^ ALSFRS-R score and ROADS score during sample collection.

^*e*^ N/A, not applicable.

### Sample collection and processing

On the day of inclusion, fresh fecal samples were collected from ALS patients and healthy controls. Samples were immediately placed in sterile fecal collection containers, transported to the laboratory, and stored at −80°C until metagenomic and metabolomics analyses were performed. Genomic DNA was isolated using the PowerSoil DNA Isolation Kit (Qiagen) according to the manufacturer’s instructions. Paired-end sequencing was performed using metagenomic shotgun sequencing on the Illumina NovaSeq X Plus platform (Novogene Co., Ltd., Beijing, China). Metabolomics analysis was subsequently performed on the Thermo QE HF/HF-X platform (Novogene Co., Ltd., Beijing, China).

### Statistical analysis

For metagenomic data, FastQC was used to check the quality of the raw sequences. KneadData (http://huttenhower.sph.harvard.edu/kneaddata) was then applied to trim adaptors and filter low-quality reads (length < 50 bp, quality value < 20, or containing N bases). The remaining high-quality sequences were used for further analysis. Taxonomic classification at the species level was performed using Kraken2. Functional annotations of gut metagenome data were performed using HUMAnN3 (20) with the ChocoPhlAn nucleotide database and UniRef90 protein database (21). Rarefaction curves, alpha diversity indices, and beta diversity (principal coordinate analysis, PCoA) were plotted using the vegan package in R. Permutational multivariate ANOVA (PERMANOVA) was performed for beta diversity analysis. To identify differentially abundant taxa, linear discriminant analysis effect size (LefSe) was used. Differentially enriched biological pathways and gene families were determined using MaAsLin2.

For metabolomics data, NovoMagic (https://magic-plus.novogene.com/) was used to perform multivariate statistical analyses. Partial least squares discriminant analysis (PLS-DA) was conducted, and model reliability was confirmed through cross-validation. The variable importance in projection (VIP) score was used to measure the contribution of metabolites to group separation. A VIP score >1.0 and *P* < 0.05 were used as the cutoff criteria for screening differential metabolites. KEGG enrichment analysis was also performed on the metabolome.

## RESULTS

### Microbial community diversity and richness

**Fig 1:**
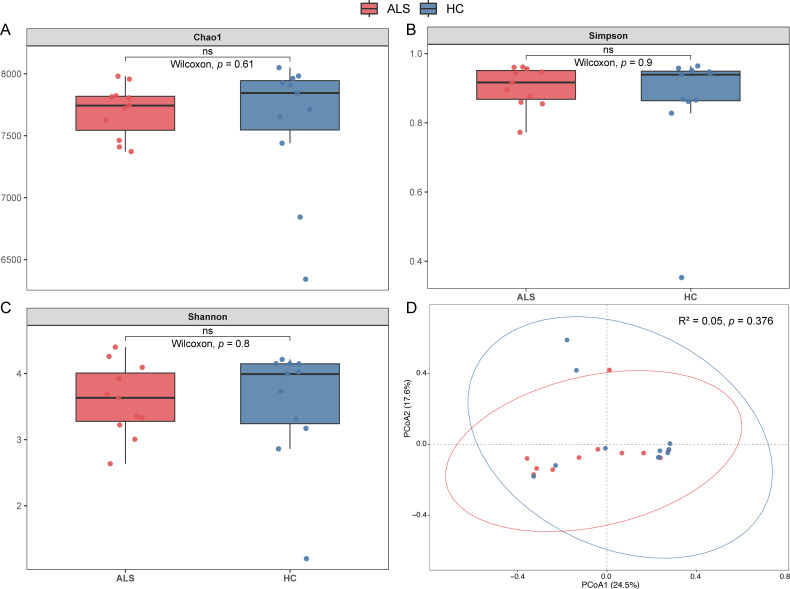
Comparison of gut microbiota diversity and community structure between ALS patients and healthy controls. (**A–C**) Alpha diversity indices of the gut microbiome in the ALS group (red, *n* = 11) and the HC group (blue, *n* = 11). Boxplots display (**A**) the Chao1 index (richness), (**B**) Simpson index (diversity), and (**C**) Shannon index (diversity). (**D**) Beta diversity analysis visualized using PCoA based on Bray–Curtis dissimilarity at the genus level.

Rarefaction analysis showed that sequencing covered the majority of species diversity in both groups, implying sufficient sequencing depth (Fig. S1). Alpha diversity (community richness and diversity) was assessed by calculating the Shannon, Simpson, and Chao1 indices. As visualized in Fig. 1, there were no significant differences in microbial richness or diversity between the ALS and HC groups (*P* > 0.05). PCoA based on Bray–Curtis dissimilarity was used to assess differences in microbial community structure. PERMANOVA analysis revealed no significant difference in global beta diversity between the two groups (*P* = 0.376).

### Taxonomic changes in the ALS microbiome

**Fig 2:**
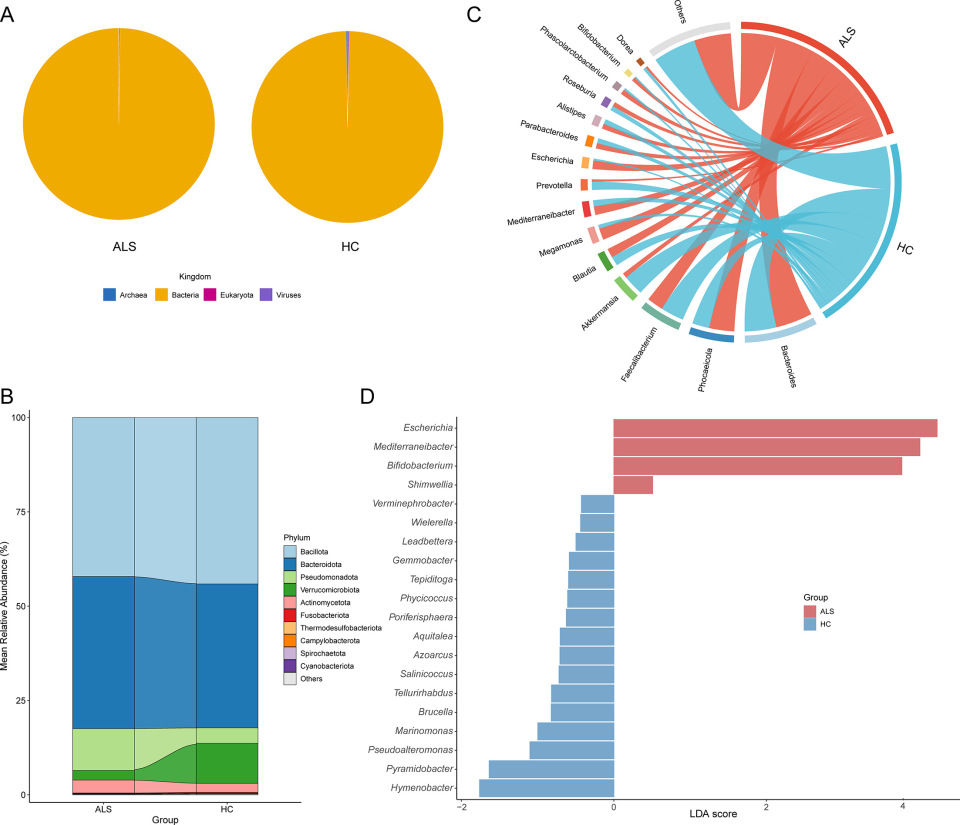
Taxonomic composition and differentially abundant taxa of the gut microbiome in ALS patients and healthy controls. (**A**) Pie charts show kingdom level in the ALS and HC. (**B**) Major phyla; less abundant (<1%) and unclassified taxa are grouped together as “other”. (**C**) Major genera; less abundant (<1%) and unclassified taxa are grouped together as “other”. (**D**) Differential genera.

Bacteria were the most abundant kingdom, representing >98% of the relative abundance in each sample, whereas Archaea, Eukaryota, and viruses were present at lower relative abundances (Fig. 2A). Organisms were classified into 44 phyla and 1,880 genera.

At the phylum level, a total of 44 phyla were identified in the two sample groups. *Bacillota* (ALS 42.14%, HC 44.08%) and *Bacteroidetes* (ALS 40.30%, HC 38.18%) were predominant in both groups. *Pseudomonadota* had a higher abundance in the ALS group (ALS 11.10%, HC 4.05%), while *Verrucomicrobiota* was more abundant in the HC group (10.63% vs 2.55%) (Fig. 2B).

Figure 2C and D shows significant differences in relative abundance at the genus level. The top 15 genera include *Bacteroides*, *Phocaeicola*, *Faecalibacterium*, *Akkermansia*, *Blautia*, *Megamonas*, *Mediterraneibacter*, *Prevotella*, *Escherichia*, *Parabacteroides*, *Alistipes*, *Roseburia*, *Phascolarctobacterium*, *Bifidobacterium*, and *Dorea*. Among these, *Bacteroides* and *Phocaeicola* were more abundant in the ALS group. Conversely, *Faecalibacterium* and *Akkermansia* showed a decreasing trend in the HC group. *Akkermansia* is a known mucin-degrading bacterium associated with intestinal barrier integrity and anti-inflammatory activity (22). *Faecalibacterium*, a major butyrate producer (23), was significantly reduced in the ALS group, potentially indicating impaired barrier function. LEFSe analysis identified *Escherichia*, *Mediturneibacter*, and *Bifidobacterium* as biomarkers for the ALS group, while *Hymenobacter* and *Pyramidobacter* were biomarkers for the HC group.

**Fig 3:**
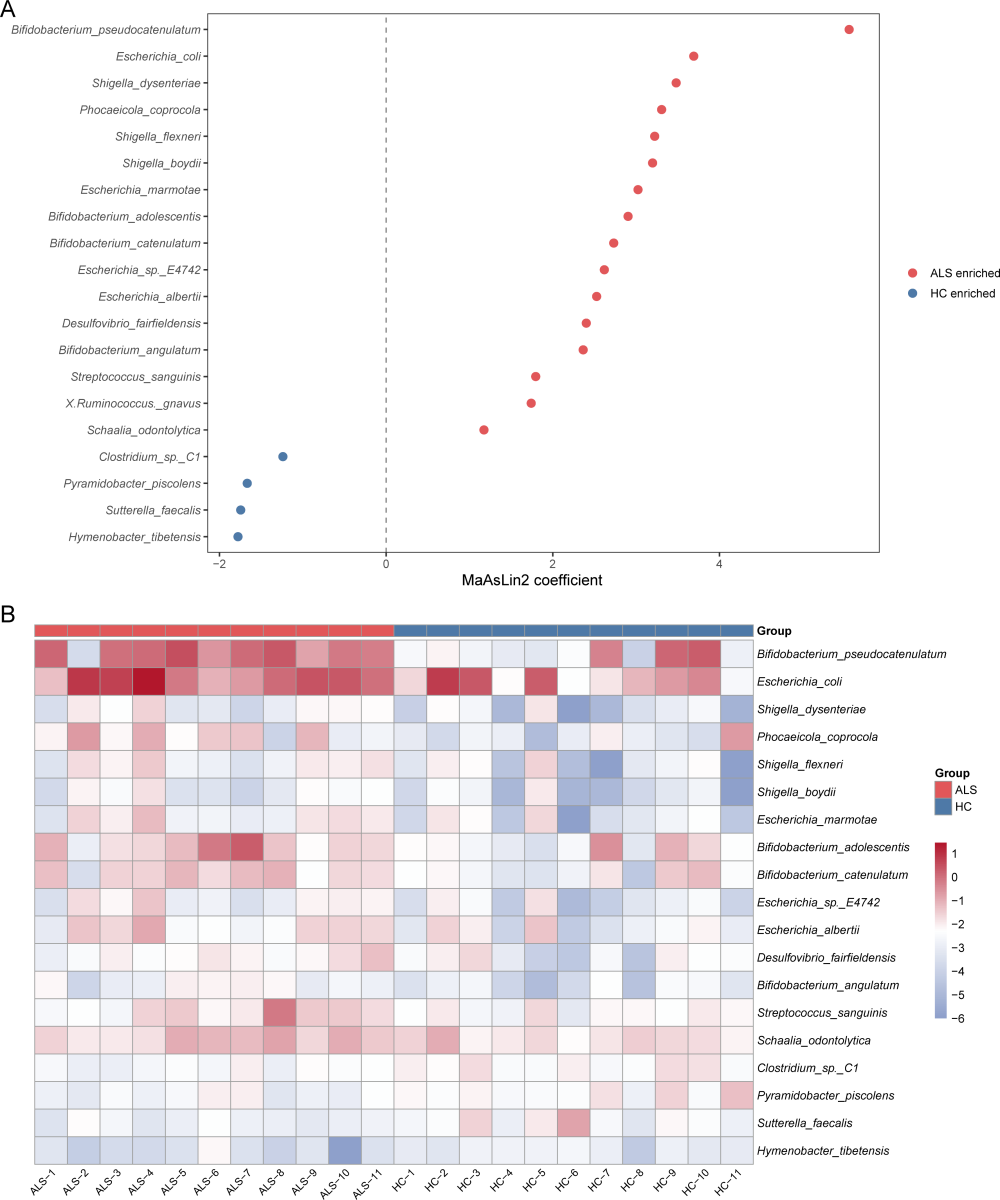
Relative abundance and differentially abundant taxa at the species level. (**A**) Identification of differentially abundant bacterial species between ALS patients and healthy controls using MaAsLin2 analysis. (**B**) Heatmap visualizing the normalized relative abundance profiles of the identified differential species across all individual samples.

At the species level (Fig. 3)**,** MaAsLin2 analysis revealed significant shifts. Compared to the HC group, several opportunistic pathogens, including *Escherichia coli*, *Shigella dysenteriae/flexneri/boydii*, and *Escherichia albertii*, were significantly increased in the ALS group. This suggests a potential for low-grade inflammation or impaired intestinal barrier function, providing an ecological advantage for these bacteria. Additionally, *Streptococcus sanguinis* and *Schaalia odontolytica*, typical oral bacteria, were elevated in the ALS gut. This strongly suggests the ectopic colonization of oral bacteria due to dysphagia, sialorrhea, or altered hygiene habits common in ALS. The enrichment of *Bifidobacterium* species may be a result of unreported probiotic intervention or a compensatory host response.

### Alterations in the gut virome

We observed a distinct enrichment of several viral genera, including *Puppervirus*, *Donellivirus*, *Pithovirus*, *Chloriridovirus*, *Tinduovirus*, and *Dhakavirus*, in ALS samples. In contrast, these taxa were either absent or present at lower relative abundances in HC samples. These findings indicate a specific shift in the viral community structure associated with ALS, hinting at a potential link between the gut virome and disease pathogenesis, although further validation is required.

### Functional gene characteristics of the ALS microbiome

To investigate the functional consequences of dysbiosis, we predicted the functional potential of the microbiome. PCA and PERMANOVA analysis (Fig. S2) showed that the pathway abundance profiles differed significantly between ALS and HC groups based on functional Bray–Curtis dissimilarities (*P* = 0.0325), revealing profound differences in the metabolic potential of the bacterial communities.

**Fig 4:**
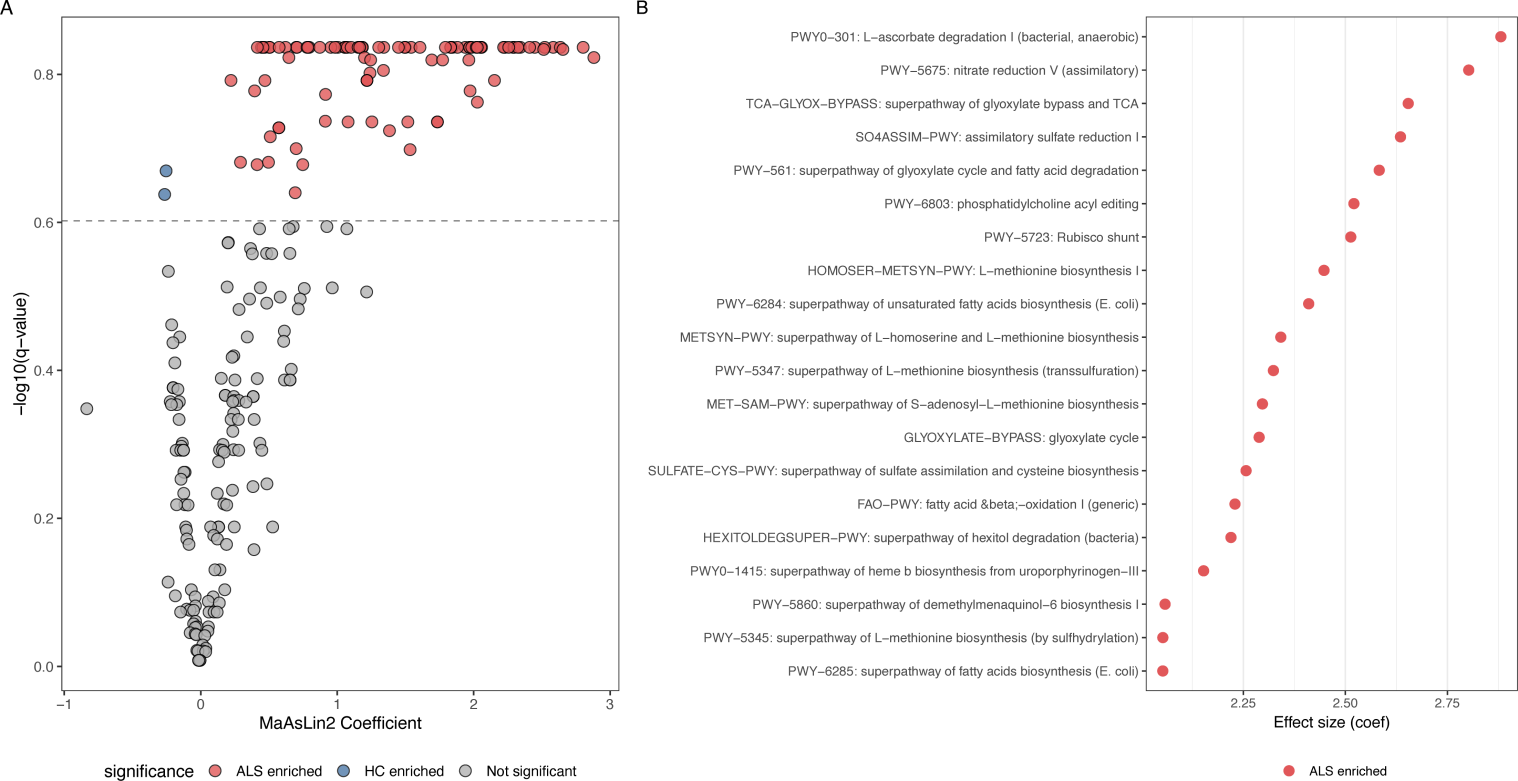
Functional alterations of the gut microbiome in ALS patients compared to healthy controls based on HUMAnN3. (**A**) Volcano plot illustrating the top 20 differential functional pathways between the ALS and HC groups identified by MaAsLin2 analysis. (**B**) Dot plot ranking the top significantly enriched metabolic pathways in the ALS microbiome based on effect size.

A total of 246 functional pathways were identified. Using MaAsLin2 (*q* < 0.25), 100 pathways were found to be differentially expressed. As shown in Fig. 4, pathways such as L-ascorbate degradation I (bacteria, anaerobic) showed the highest effect size, suggesting an enhanced microbial capacity to degrade vitamin C, a potent antioxidant, in the gut of ALS patients. Furthermore, pathways involved in central energy metabolism, such as the TCA cycle and glyoxylate bypass, were significantly upregulated. We also observed distinct alterations in lipid and amino acid metabolism, characterized by the enrichment of fatty acid biosynthesis/oxidation pathways and L-methionine biosynthesis pathways. These functional shifts suggest that the ALS-associated microbiome may contribute to pathology by exacerbating oxidative stress and disrupting metabolic homeostasis. Notably, species contribution analysis (Fig. S3) revealed that these differential pathways are primarily driven by *Escherichia coli* and *Klebsiella pneumoniae*.

### Fecal metabolomics changes in ALS

**Fig 5:**
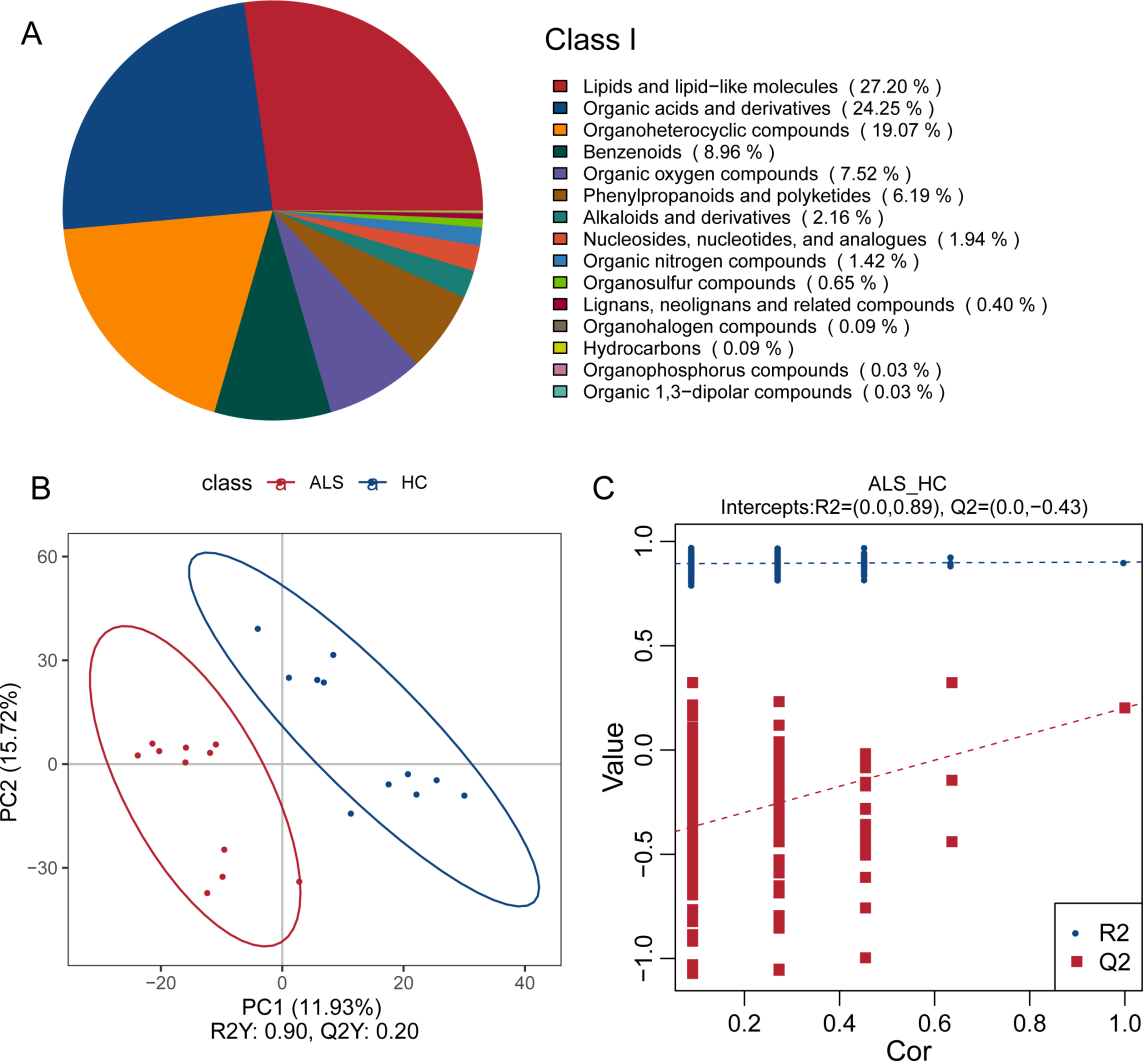
Overview of the fecal metabolome composition and PLS-DA analysis between ALS patients and healthy controls. (**A**) Pie chart illustrating the chemical classification of identified metabolites at Class I level. (**B**) PLS-DA analysis and (**C**) permutation test plot validating the robustness of the PLS-DA model. The intercept of the Q2 regression line is less than 0 (Q2 intercept = −0.43), indicating that the PLS-DA model is reliable and not overfitted.

LC-MS metabolomics analysis identified 6,408 metabolites from the 22 fecal samples. The identified metabolites were predominantly lipids and lipid-like molecules (27.2%), organic acids and their derivatives (24.25%), and organic heterocyclic compounds (19.07%) (Fig. 5A). PLS-DA analysis demonstrated a clear separation between ALS and HC groups, confirming distinct fecal metabolomic profiles (Fig. 5B).

**Fig 6:**
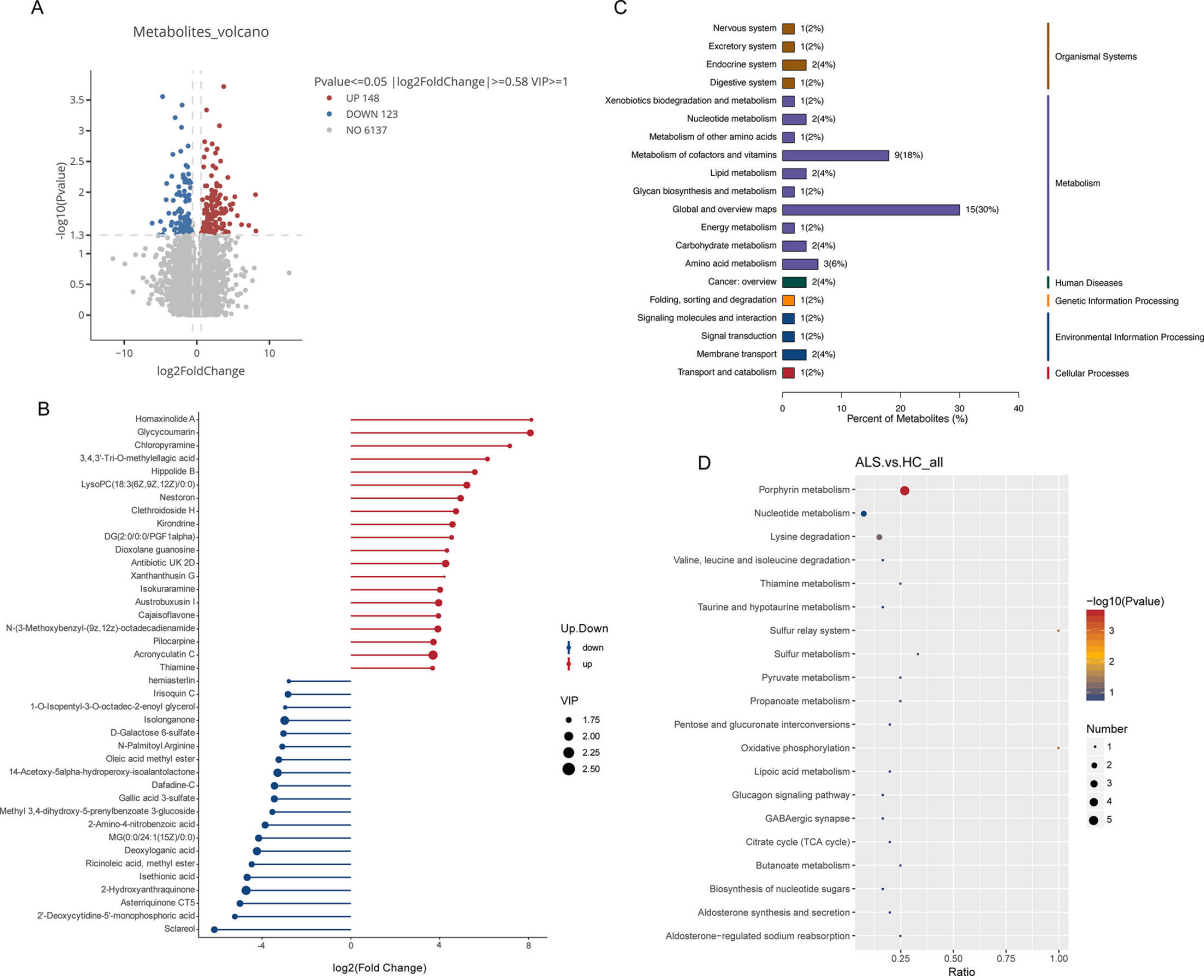
Identification and functional analysis of differentially expressed metabolites in the feces of ALS patients. (**A**) Volcano plot visualizing the differential metabolites between ALS patients and healthy controls. (**B**) Lollipop chart displaying the top differentially expressed metabolites ranked by log2 (fold change). (**C**) KEGG pathway classification of the differential metabolites. (**D**) KEGG pathway enrichment analysis bubble plot.

Using VIP > 1.0 and *P* < 0.05, we identified 271 differentially expressed metabolites (148 upregulated, 123 downregulated) (Fig. 6A). Figure 6B highlights key metabolites. In the ALS group, upregulated metabolites included potential compensatory neuroprotective compounds like glycycoumarin (24), kirondrine (25), and cajaisoflavone (26). However, we also observed elevated LysoPC(18:3), a lysophosphatidylcholine associated with cell membrane damage and inflammation (27), suggesting decreased neuronal membrane stability. Elevated thiamine levels likely reflect vitamin supplementation. Upregulation of lipids and nucleoside analogs suggests involvement of lipid signaling and nucleic acid metabolism in ALS pathology.

Conversely, significant decreases were observed in metabolites related to energy metabolism and endogenous protection (28). Decreases in D-galactose 6-sulfate (29), deoxyloganic acid, and methyl 3,4-dihydroxy-5-prenylbenzoate 3-glucoside (30) may reflect impaired glucose metabolism and insufficient energy supply. Reductions in N-palmitoyl arginine (31), ricinoleic acid (32), and methyl ester lipids (33) suggest abnormal lipid synthesis, possibly linking to impaired myelin maintenance. Furthermore, decreases in sclareol (34), isethionic acid (35), and 2-hydroxyanthraquinone (36)—natural products with antibacterial and antioxidant activities—indicate weakened endogenous protective mechanisms and exacerbated oxidative damage. Decreases in 2-deoxycytidine-5-monophosphoric acid, as a nucleotide metabolite, may reflect decreased DNA synthesis/repair capacity, associated with neuronal apoptosis or degeneration.

KEGG enrichment analysis (Fig. 6C and D) identified significant dysregulation in porphyrin metabolism, nucleotide metabolism, lysine degradation, and multiple energy-related pathways such as oxidative phosphorylation and the citrate cycle (TCA cycle). These results underscore widespread disruptions in energy homeostasis, amino acid metabolism, and neuroendocrine regulation in ALS.

### Correlations between gut dysbiosis, metabolic alterations, and clinical severity

To explore the clinical relevance of our multi-omics findings, we performed Spearman correlation analyses between key differential features and the ALSFRS-R scores.

As shown in Fig. S4A, several bacterial genera were negatively correlated with ALSFRS-R scores, such as *Verminephrobacter* (*r* < −0.8), suggesting that their expansion tracks with disease severity. Interestingly, *Escherichia* showed a weak positive correlation trend, though not statistically significant in this analysis.

In the metabolomic analysis (Fig. S4B), N-palmitoyl arginine—which we found to be significantly downregulated in the ALS group (Fig. 6)—exhibited the strongest positive correlation with ALSFRS-R scores (*r* > 0.7). This indicates that the depletion of this lipid metabolite is closely linked to worse functional outcomes in patients. Conversely, methyl 3,4-dihydroxy-5-prenylbenzoate 3-glucoside showed a negative correlation with the clinical score.

Furthermore, to decipher the microbe-metabolite interplay, we constructed a correlation heatmap (Fig. S4C). Notably, N-palmitoyl arginine showed a significant positive correlation with *Bifidobacterium* (*P* < 0.05). Given that both *Bifidobacterium* and N-palmitoyl arginine are linked to better health states, this association suggests a potential beneficial axis where specific gut commensals may influence lipid homeostasis relevant to neuronal integrity.

## DISCUSSION

In this study, we performed the first integrative multi-omics analysis of the gut microbiome and metabolome in Chinese patients with ALS. While global diversity indices did not show significant differences, we identified profound shifts in taxonomic composition, functional gene potential, and metabolic profiles. Our findings reveal a gut ecosystem characterized by a depletion of anti-inflammatory commensals, an expansion of opportunistic pathogens, and a functional shift toward oxidative stress exacerbation and metabolic dysregulation. These results support the hypothesis that gut dysbiosis acts as a driving force in ALS pathology via the gut-brain axis (37).

A hallmark finding of our study was the significant depletion of *Faecalibacterium* in ALS patients. *Faecalibacterium* is a major producer of butyrate (38), an anti-inflammatory SCFA that maintains tight junctions and modulates immune responses (39). The loss of these protective genera aligns with previous observations in animal models (37) and Western ALS cohorts (40), suggesting that compromised gut barrier function is a universal feature of ALS, independent of geographic background. Although we excluded participants with recent antibiotic use, strict control over over-the-counter probiotic supplements was challenging. If the increase is due to unreported supplementation, it suggests that standard probiotics might not be sufficient to reverse the core dysbiosis (e.g., the expansion of *E. coli* and vitamin C degradation) observed in these patients. Alternatively, if this is a host compensatory response, the upregulation of *Bifidobacterium* (known for anti-inflammatory properties) might represent an attempt by the gut ecosystem to counteract the inflammation driven by pathobionts like *Escherichia*. This implies that the gut microbiome in ALS is in a state of dynamic conflict between pathogenic drivers and restorative responders.

Concomitant with the loss of beneficial bacteria, we observed an enrichment of *Pseudomonadota*, specifically the genus *Escherichia* and species *E. coli* and *Shigella* spp. These gram-negative bacteria are potent sources of LPS, which can promote microglial activation and motor neuron death (40). Furthermore, our study identified a significant increase in oral-associated bacteria, such as *Streptococcus sanguinis* and *Schaalia odontolytica*, in the fecal samples of ALS patients, consistent with previous observations (41). This likely reflects the “oral-gut axis” dysfunction; dysphagia and sialorrhea (excessive drooling) common in ALS may alter the oral microbiota and facilitate the ectopic colonization of oral bacteria in the gut. This suggests that clinical management of oral hygiene and swallowing function might impact gut health in ALS.

A key novelty of our study lies in the functional metagenomic analysis. We identified a striking enrichment of the L-ascorbate (vitamin C) degradation pathway, primarily driven by *E. coli*. Vitamin C is a critical antioxidant in the central nervous system (42). The enhanced microbial capacity to degrade vitamin C suggests that the dysbiotic gut microbiome may actively deplete host antioxidant reserves, thereby exacerbating the oxidative stress that is central to ALS pathogenesis. This provides a specific, actionable mechanism linking gut bacteria to neurodegeneration. Additionally, pathways related to the TCA cycle and glyoxylate bypass were upregulated. Given that ALS is characterized by systemic hypermetabolism, the gut microbiome may be shifting its metabolic activity to compensate for, or contribute to, the host’s altered energy demands.

Our metabolomic analysis corroborated the metagenomic findings, revealing widespread disruptions in energy and lipid metabolism. We observed elevated levels of LysoPC, a marker of inflammation and membrane breakdown (43). High LysoPC levels have been associated with demyelination and neuroinflammation (44), suggesting that gut-derived lipid metabolites may directly affect neuronal membrane stability. Conversely, we noted a downregulation of antioxidant and neuroprotective metabolites, such as bile acid derivatives and specific amino acid metabolites. The enrichment of pathways involving porphyrin metabolism and oxidative phosphorylation in the metabolome further underscores the systemic metabolic stress present in ALS. The increase in certain potentially neuroprotective compounds (e.g., glycycoumarin) in the ALS group may represent a compensatory but insufficient response to the ongoing neurodegenerative process.

Our study has several limitations. First, the sample size (*n* = 11 per group) is relatively small, which may limit the statistical power to detect subtle changes in diversity. However, the use of high-resolution shotgun metagenomics compensates for this by providing deep functional insights. Second, this is a cross-sectional study, preventing us from establishing causality; it remains unclear whether dysbiosis causes ALS progression or results from the disease (e.g., due to immobility or dietary changes). Third, while we matched for age and sex, strict control of long-term dietary habits was not feasible, which may influence the microbiome. Future studies should employ longitudinal designs with larger cohorts and validate the function of specific viral and bacterial taxa in animal models.

In conclusion, this study characterizes the gut microbiome and metabolome of Chinese ALS patients, revealing a distinct dysbiotic profile marked by the loss of *Akkermansia*, enrichment of *Escherichia*, and the intrusion of oral taxa. Critically, we identified a functional link between the microbiome and disease pathology, specifically through the microbial degradation of antioxidants (vitamin C) and the dysregulation of lipid and energy metabolism. These findings highlight the gut-brain axis as a potential therapeutic target, suggesting that interventions aimed at restoring *Akkermansia* levels or inhibiting microbial vitamin C degradation could offer new avenues for ALS management.

## Data Availability

The sample and sequence data obtained in this study have been submitted to the NCBI BioSample and Sequence Read Archive (SRA) under BioProject accession number PRJNA1395841.

## SUPPLEMENTAL MATERIAL

Supplemental figures spectrum.00609-26-s0001.pdf Fig. S1 to S4.

ASM does not own the copyrights to Supplemental Material that may be linked to, or accessed through, an article. The authors have granted ASM a non-exclusive, world-wide license to publish the Supplemental Material files. Please contact the corresponding author directly for reuse.
