# Supplementary material for: Integrated multi-omics analysis reveals gut dysbiosis and altered energy metabolism in Chinese ALS patients
Source: Microbiol Spectr. 2026 Apr 30;14(6):e00609-26. doi: 10.1128/spectrum.00609-26 (PMC13227962; doi:10.1128/spectrum.00609-26)
Supplement: Supplemental figures — Fig. S1 to S4. [file spectrum.00609-26-s0001.pdf]

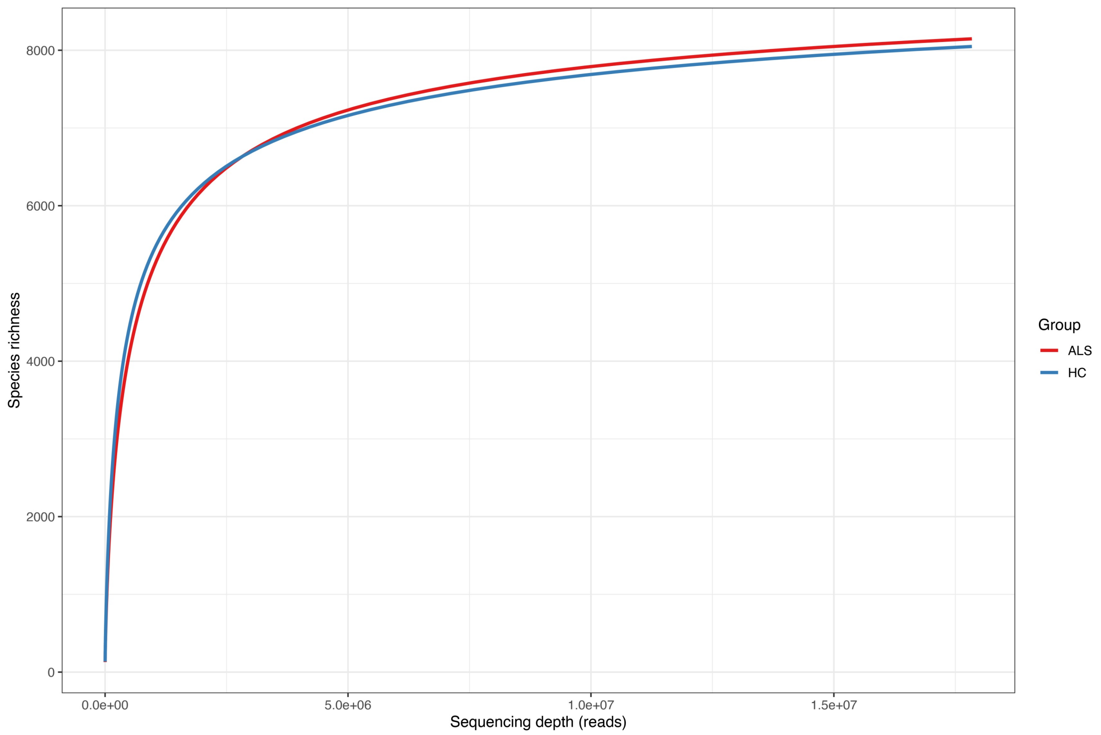

Figure S1. Rarefaction curves of genus level.

Rarefaction analysis showed that the species richness in each group approached saturation, implying that the current sequencing depth covered the largest species diversity

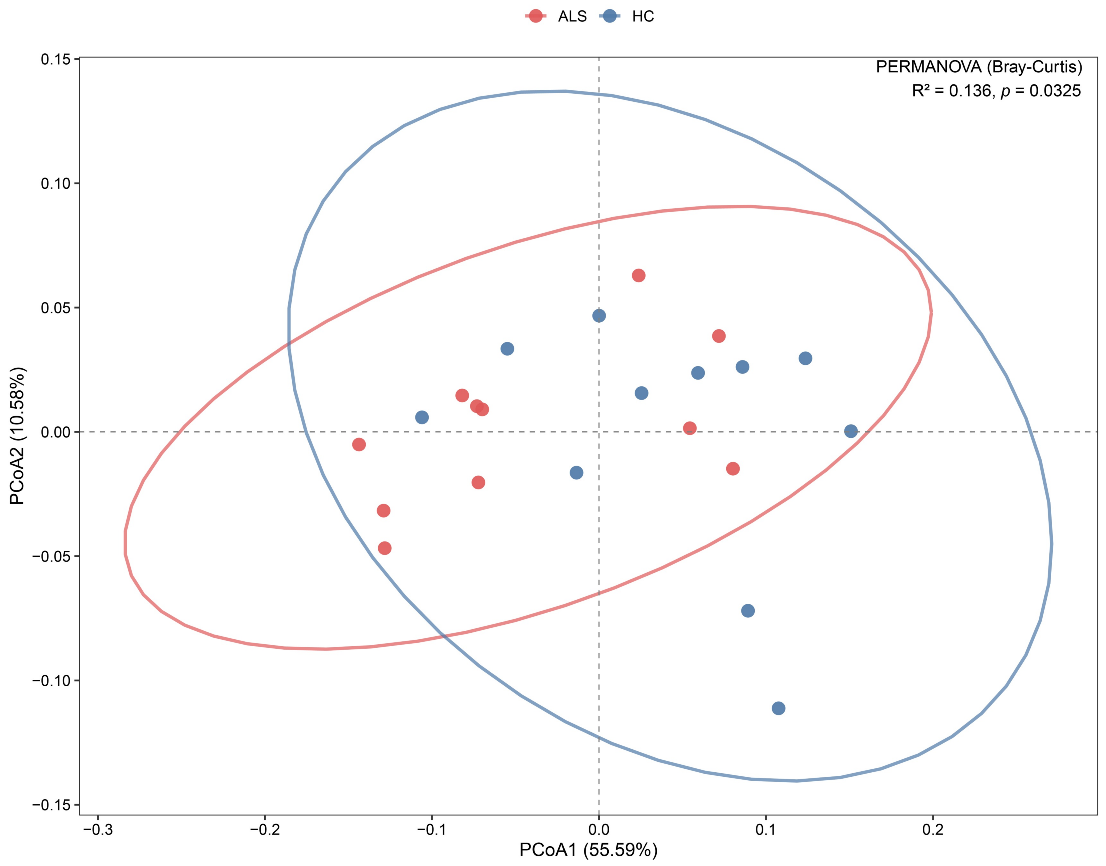

Figure S2. PCoA analysis on the gut microbiome functional profiles of ALS and HC.

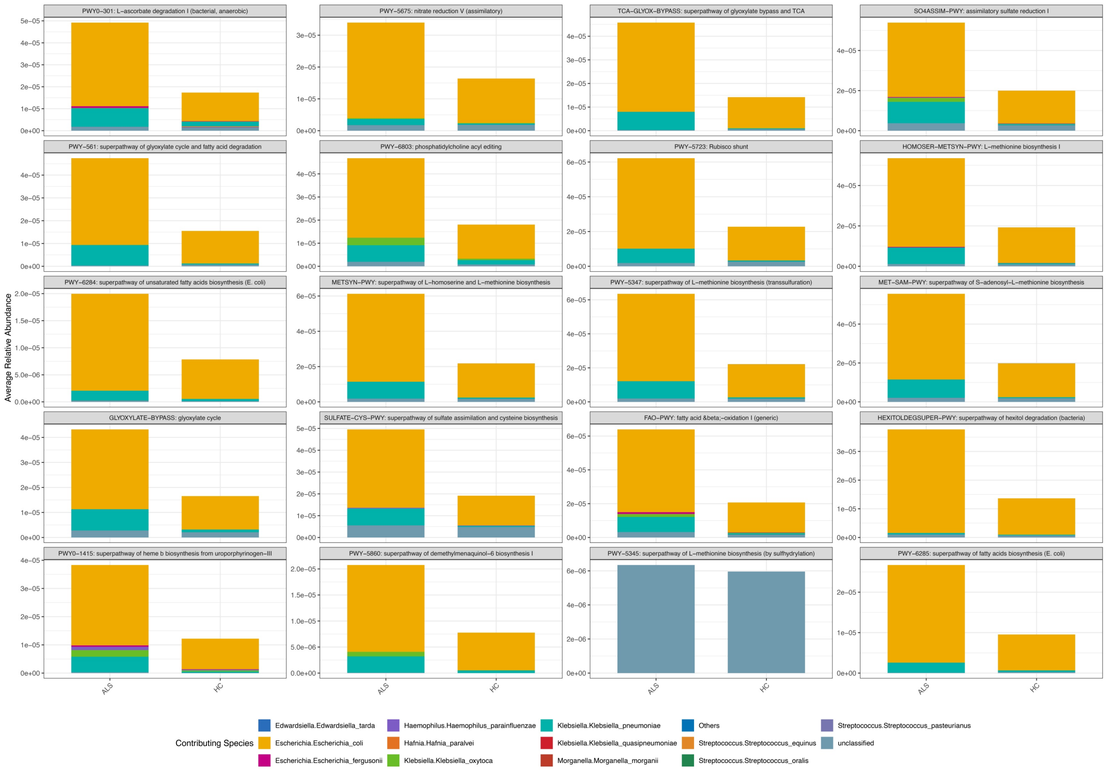

Figure S3. Taxonomic stratification of the top differentially abundant functional pathways.

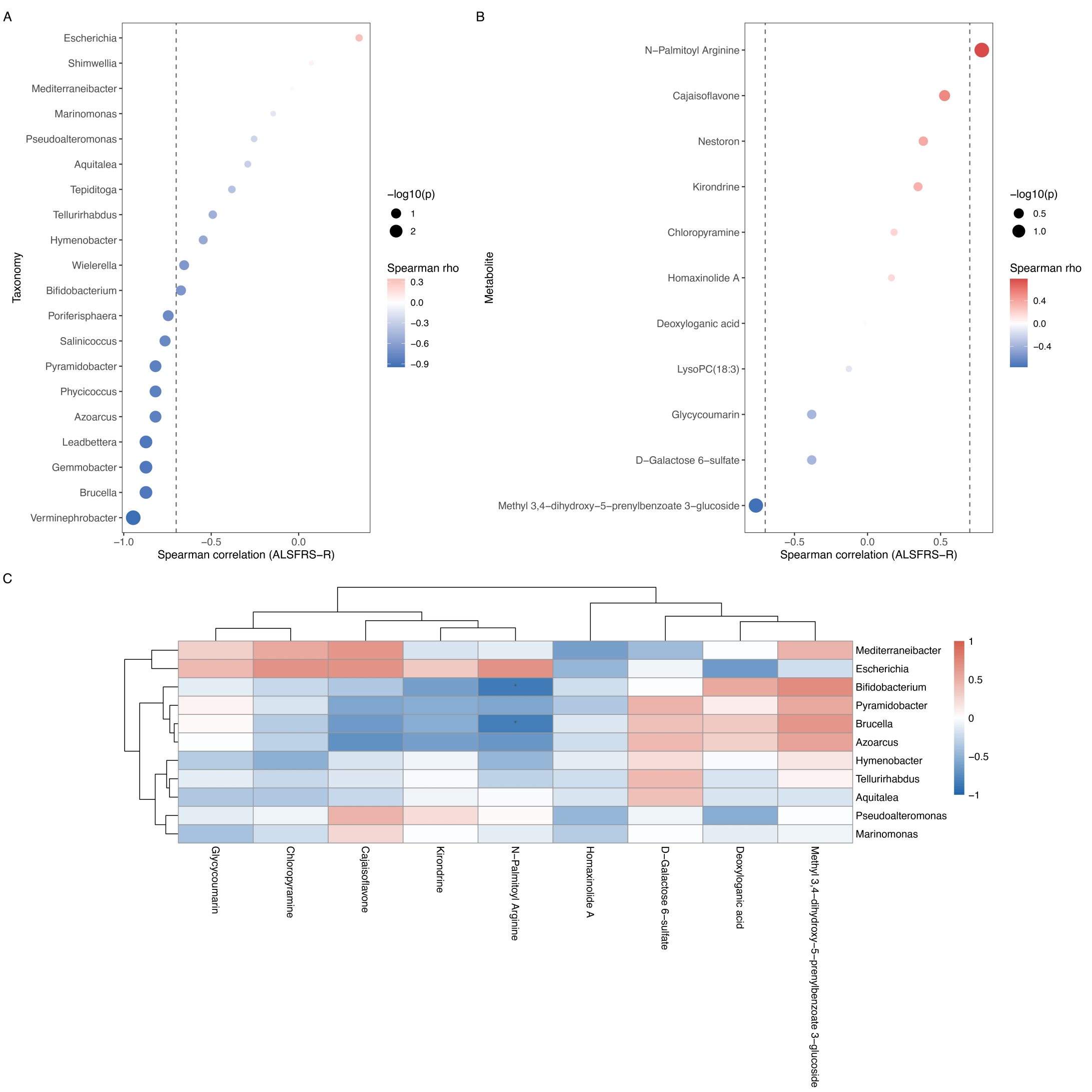

Figure S4. Integrative correlation analysis linking the gut microbiome, fecal metabolome, and clinical severity in ALS patients.

(A) Spearman correlations between the relative abundances of differentially abundant bacterial genera and ALS Functional Rating Scale–Revised (ALSFRS-R) scores. (B) Spearman correlations between differentially abundant fecal metabolites and ALSFRS-R scores. (C) Spearman correlations between differentially abundant bacterial genera and fecal metabolites.Asterisks denote statistically significant associations ($P < 0.05$).
